# Purulent bullous epidermal necrolysis: A potential new clinical pattern of drug eruption

**DOI:** 10.3892/etm.2013.944

**Published:** 2013-02-01

**Authors:** LUN-FEI LIU, JIAN-LIANG YAN, JIAN-YOU WANG, JI-SU CHEN, SUI-QING CAI, LI-MIN LAO, MIN ZHENG

**Affiliations:** Department of Dermatology, Second Affiliated Hospital, School of Medicine, Zhejiang University, Hangzhou 310009, P.R. China

**Keywords:** drug eruption, purulent bulla, epidermal necrolysis

## Abstract

Drug eruption is a major problem of adverse drug reactions and may present as variform clinical manifestations. Toxic epidermal necrolysis (TEN) and acute generalized exanthematous pustulosis (AGEP) are relatively rare severe drug eruptions. It has rarely been reported that AGEP overlaps or mimics TEN, while no purulent bullous epidermal necrolysis has been reported. The present study reports a rare case of an adult female patient with the clinical manifestations of purulent bulla and epidermal necrolysis caused by drug ingestion. The case is discussed to reveal whether a new clinical pattern of drug eruption has been identified.

## Introduction

Adverse drug reactions (ADRs) are a big challenge in drug therapy, with cutaneous drug reactions accounting for a large proportion. The clinical manifestations of drug eruptions are highly variable. It is critical to recognize and deal with severe cutaneous ADRs (SCADRs) as rapidly as possible as they are able to cause life-threatening diseases, including Stevens-Johnson syndrome, toxic epidermal necrolysis (TEN), generalized bullous fixed drug eruption, acute generalized exanthematous pustulosis (AGEP) and drug reactions with eosinophilia and systemic symptoms (1,2).

The present study describes the rare case of a female patient with medication-triggered epidermal necrolysis and purulent bulla throughout her body, whose drug eruption was successfully cured. To the best of our knowledge, no drug eruption with purulent bulla and epidermal necrolysis has previously been documented, and therefore the present study is the first case report of its kind.

## Case report

A 51-year-old female was referred to the Second Affiliated Hospital, Hangzou, China, as a result of drug anaphylaxis, which developed 3 days prior to admittance and became aggravated rapidly. The present study was conducted in accordance with the declaration of Helsinki and with approval from the Ethics Committee of the Second Affiliated Hospital, School of Medicine, Zhejiang University. Written informed consent was obtained from the patient. The patient suffered a scalp trauma in a traffic accident and was presented to a local hospital eight days prior to her referral to the Second Affiliated Hospital, Hangzou. A single dose of 1500 IU tetanus antitoxin (TAT) was prescribed immediately following debridement. Subsequently, 8 g/day of intravenus sulbenicillin was administered for a total of 5 days. Following this, the patient suffered from moderate pruritus with a sudden occurrence of generalized erythema and numerous easily ruptured pustules the size of peas, however she had neither pain nor fever. Although 10 mg/day of intravenus dexamethasone was administered, the skin rash progressed rapidly with epidermal necrolysis, generalized purulent bulla and erosion of the oral and vulval mucosa in the succeeding 3 days. The patient suffered intense causalgia. A patient history revealed no drug or food allergies and no history of personal or familial psoriasis.

**Figure 1:**
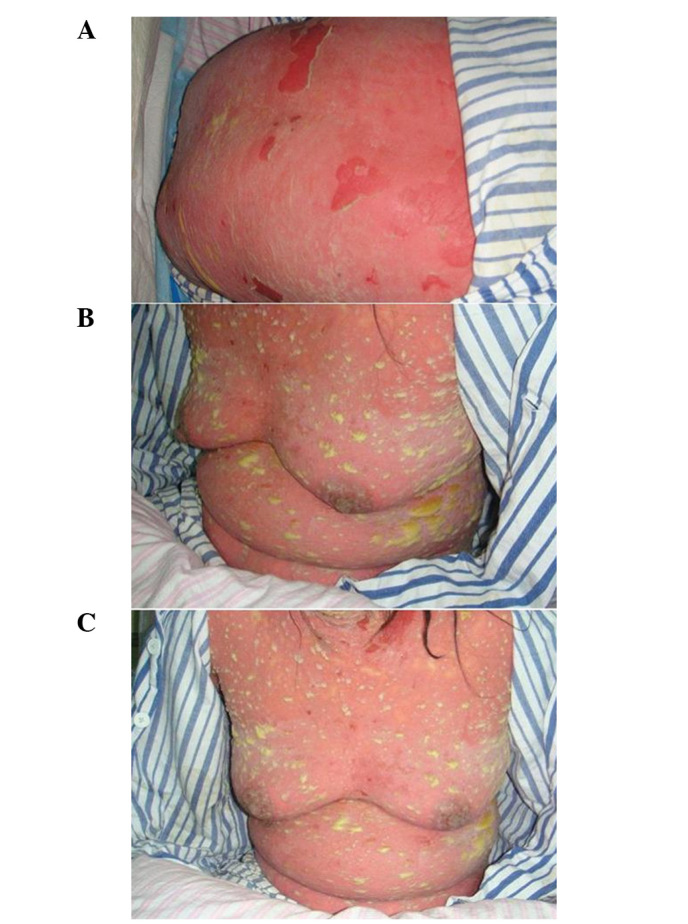
Diffuse bright red area, non-follicular pustules and generalized pulurent bulla with epidermal necrolysis in the trunk. (A) Back; (B) side; and (C) front views.

On admission after referral, the patient was alert and cooperative. A physical examination showed no significant abnormalities, with the exception of the skin eruptions and severe pitting edema on the lower extremities. The patient developed diffuse bright red areas, non-follicular pustules and a generalized purulent bulla all over the body, with epidermal necrolysis and the detachment of 46% of the body surface area (BSA; Fig. 1). There was erosion in the oral and vulval mucosa and Nikolsky’s sign was positive.

Laboratory studies revealed leukocytosis (23.3×10^9^/l) and neutrophilia (22.5×10^9^/l). With the exception of low albumin (3.03 mg/dl) and high C-reactive protein (CRP; 287.3 mg/l) levels, the other blood chemical examinations were normal. The blood and pus cultures were negative for bacteremia. The search for specific IgE antibodies to inhalants and food was performed by UniCAP RAST (Phadia, Uppsala, Sweden) and was negative. A chest X-ray revealed no abnormal imaging results. A skin biopsy was not performed as the idea was rejected by the patient and her dependents, meaning that a written informed consent was not obtainable. The clinical impression obtained was one of a drug eruption with purulent bulla and epidermal necrolysis. The patient was treated with intravenous methylprednisolone and additional 0.4 g/kg/day intravenous immunoglobulin (IVIG) for 5 consecutive days. The quantities of purulent bulla markedly decreased and the causalgia lessened on the third day of hospitalization. The purulent bulla and epidermal necrolysis disappeared on the fifth day. The dosage of methylprednisolone was gradually decreased and the laboratory values were also improved in the following days (Table I).

## Discussion

The patient was diagnosed with a drug eruption with purulent bulla and epidermal necrolysis based on the following criteria: i) the symptoms began 5 days after starting the TAT and sulbenicillin treatments; ii) the presence of a generalized purulent bulla, epidermal detachment of a large section of the body surface area, erosions of the mucous membranes and a positive result for Nikolsky’s sign; iii) the culture of the purulent bulla content was negative for bacteria; iv) the symptoms were not attributable to bacterial or viral infections; and v) the clinical signs and laboratory abnormalities were responsive to corticosteroids and IVIG. To date, pustular drug exanthema, which refers in particular to AGEP (3), is not common. Furthermore, TEN accompanied by AGEP has rarely been reported (4). In the present case, an acute onset of widespread erythema, pustules and purulent bulla, leukocytosis, neutrophilia, a large area of epidermal necrolysis, erosion of the mucous membranes of the cavitas oris and labium minus and a positive Nikolsky’s sign over the whole body were present following drug administration. The clinical features appeared similar to AGEP and TEN to a certain extent, but the widespread purulent bulla, which were >5 mm in diameter, emerged on the disease onset and were not confluent with adjacent pustules, meant that the diagnosis differed from that of AGEP. As there has been no previously reported drug eruption displaying purulent bulla and epidermal necrolysis, we suspect that this case represents a new clinical pattern and have therefore named it purulent bullous epidermal necrolysis.

A differential diagnosis should be made to distinguish the condition of the patient in the present study from that of other bullous diseases. Bullous impetigo may be excluded by the negative results of the pus bacterial culture and the good response to corticosteroids. It is unfortunate that a skin biopsy was not performed, but from the typical clinical manifestation observed, a diagnosis of other bullous diseases, including bullous fixed drug eruption, drug-induced bullous pemphigus or drug-induced bullous pemphigoid, may also be excluded.

It has been reported that AGEP is mostly caused by a reaction to antibiotics, particularly the β-lactams and macrolides (5). TEN is usually caused by antibiotics, non-steroidal anti-inflammatory drugs (NSAIDs), anticonvulsants and antipodagrics (6,7). In the present study, the patient suffered a drug eruption following the administration of TAT and sulbenicillin. Although a previous study has reported TEN caused by TAT (8), no reports of TAT causing purulent eruptions or sulbenicillin-related TEN or purulent eruptions have been documented. It is difficult to determinine whether a particular medication is able to cause a specific eruption, particularly when the patient is taking several drugs, due to the lack of sensitivite and specific tests. Although drug provocation testing is the best tool for dertermining the causal drug of a non-immediate allergic reaction, it is dangerous and contraindicated in severe cases, including those of AGEP and TEN (9,10). In the present study the patient refused any attempt to trace the causal drug.

Physicians who prescribe TAT or sulbenicillin should be aware of this rare potential life-threatening complication.

## Figures and Tables

**Table I:** Laboratory values and clinical characteristics at 1, 2, 4, 8, 12 and 20 days post-admission.

	Day					
	
Clinical characteristics	1	2	4	8	12	20
Body temperature (°C)	37.4	37.7	36.9	37	37.1	37
White blood cell count/mm^3^	23.3	17.7	16.1	10.2	9.7	8.5
Eosinophils/mm^3^	0.3	0.2	0.1	0.1	0.1	0.1
Neutrophils/mm^3^	22.5	17	15.1	8.3	8	6
Aspartate aminotransferase (IU/l)	-	19	59	20	30	13
Alanine aminotransferase (IU/l)	-	32	65	87	51	20
Albumin (mg/dl)	-	3.03	2.39	2.87	3.03	3.52
C-reaction protein (mg/l)	-	287.3	175.7	57.6	22.9	3.5
Prednisolone (mg/day)	80	160	120	80	60	28
